# Consumption of a high-fat meal containing cheese compared with a vegan alternative lowers postprandial C-reactive protein in overweight and obese individuals with metabolic abnormalities: a randomised controlled cross-over study

**DOI:** 10.1017/jns.2015.40

**Published:** 2016-02-09

**Authors:** Elieke Demmer, Marta D. Van Loan, Nancy Rivera, Tara S. Rogers, Erik R. Gertz, J. Bruce German, Angela M. Zivkovic, Jennifer T. Smilowitz

**Affiliations:** 1 Department of Nutrition, University of California Davis, Davis, CA, USA; 2 USDA/ARS Western Human Nutrition Research Center, Davis, CA, USA; 3 Foods for Health Institute, University of California, Davis, CA, USA; 4 Department of Food Science & Technology, University of California, Davis, CA, USA

**Keywords:** Dairy products, Inflammation, Metabolic syndrome, Obesity, Palm oil, Postprandial metabolism, Vegan diets, CH, cheese, CRP, C-reactive protein, HOMA-IR, homoeostasis model assessment of insulin resistance, iAUC, incremental AUC, MCP-1, monocyte chemotactic protein-1, SAA, serum amyloid-A, sICAM, soluble intracellular adhesion molecule, VA, vegan alternative, WC, waist circumference

## Abstract

Dietary recommendations suggest decreased consumption of SFA to minimise CVD risk; however, not all foods rich in SFA are equivalent. To evaluate the effects of SFA in a dairy food matrix, as Cheddar cheese, *v*. SFA from a vegan-alternative test meal on postprandial inflammatory markers, a randomised controlled cross-over trial was conducted in twenty overweight or obese adults with metabolic abnormalities. Individuals consumed two isoenergetic high-fat mixed meals separated by a 1- to 2-week washout period. Serum was collected at baseline, and at 1, 3 and 6 h postprandially and analysed for inflammatory markers (IL-6, IL-8, IL-10, IL-17, IL-18, TNFα, monocyte chemotactic protein-1 (MCP-1)), acute-phase proteins C-reactive protein (CRP) and serum amyloid-A (SAA), cellular adhesion molecules and blood lipids, glucose and insulin. Following both high-fat test meals, postprandial TAG concentrations rose steadily (*P* < 0·05) without a decrease by 6 h. The incremental AUC (iAUC) for CRP was significantly lower (*P* < 0·05) in response to the cheese compared with the vegan-alternative test meal. A treatment effect was not observed for any other inflammatory markers; however, for both test meals, multiple markers significantly changed from baseline over the 6 h postprandial period (IL-6, IL-8, IL-18, TNFα, MCP-1, SAA). Saturated fat in the form of a cheese matrix reduced the iAUC for CRP compared with a vegan-alternative test meal during the postprandial 6 h period. The study is registered at clinicaltrials.gov under NCT01803633.

One independent risk factor for CVD is postprandial inflammation, or inflammation in the hours immediately following a meal (for a review, see Burdge & Calder^(^^1^^)^). Consumption of a meal promotes a transient inflammatory response, the intensity and duration of which is determined by the composition of the meal (for a review, see Margioris^(^^2^^)^). A high-fat meal increases TAG- rich lipoproteins, a phenomenon known as postprandial lipaemia, which is linked to CVD risk^(^^3^^)^. Both postprandial inflammatory and lipaemic responses contribute to a chronic pro-inflammatory state observed in metabolically abnormal individuals, termed metabolic inflammation, which further exacerbates CVD risk (for a review, see Margioris^(^^2^^)^). Observational studies have linked saturated fat consumption with increased risk for CVD development^(^^4^^,^^5^^)^. The specific effects of varying fatty acid composition have been investigated extensively, with the latest consensus purporting that MUFA and PUFA are beneficial whereas SFA and *trans*-fats are deleterious in terms of plasma lipid profiles and CVD risk (for a review, see Erkkilä *et al.*^(^^6^^)^). Decreasing SFA intake has been the cornerstone of dietary recommendation for several decades, including the current Dietary Guidelines for Americans 2010^(^^7^^)^. However, decreasing SFA intake may not lower CVD risk in a subset of the population^(^^8^^)^. In fact, a recent meta-analysis of twenty-two prospective studies found that consumption of certain dairy foods that are high in SFA, specifically cheese, was found to be inversely associated with CVD risk^(^^9^^)^. The postprandial effect of dairy foods is more than the sum of its parts. When analysed individually, dairy-derived proteins^(^^10^^)^, SCFA^(^^11^^)^, bacteria in fermented dairy products (for a review, see Settanni & Moschetti^(^^12^^)^), and milk fat globule membrane^(^^13^^)^, have shown favourable or neutral effects on CVD risk. However, little is known about the combination of components in cheese and their interactions, known as the food matrix (for a review, see Jacobs *et al.*^(^^14^^)^), on postprandial lipaemia and inflammation. The majority of studies examining postprandial inflammation in response to high-fat dietary challenges compared SFA with MUFA and PUFA, but not with other high-SFA foods that varied in fatty acid composition^(^^15^^,^^16^^)^. Additionally, the consumption of SFA from dairy products in the context of a complex matrix such as cheese rather than as just milk, cream or butter have not been examined. This is surprising considering that cheese is the primary source of SFA in the American diet^(^^17^^)^. A recent study documented that approximately 5 % of US adults are vegetarian, of whom about half are vegan^(^^18^^)^. These numbers have doubled from 2009 to 2011. The Dietary Guidelines Advisory Committee recently noted that a diet higher in plant-based foods is more health promoting than the current US diet^(^^19^^)^. This is echoed in a position paper from the Academy of Nutrition and Dietetics reporting that appropriately planned plant-based diets may help prevent and treat certain chronic diseases^(^^20^^)^. While there is research supporting a plant-based diet for optimal health, this type of dietary pattern can still include plant-based saturated fats, such as palm oil. Yet the postprandial effects of high-fat meals containing SFA from plant sources (i.e. palm oil) have not been examined in human subjects. In mice, consumption of a diet enriched with palm oil induced the highest levels of inflammation compared with other lipids tested, including milk fat^(^^21^^)^. Given the increasing interest in plant-based diets by the public, this study set out to address the gap in knowledge about the effects of dietary saturated fat from plant sources *v*. from dairy sources on postprandial inflammation. Specifically, the aim of this study was to determine the effects of consuming two mixed isoenergetic meals high in SFA from cheese compared with a non-dairy cheese alternative plus palm oil on postprandial inflammatory and metabolic markers. We hypothesised that consumption of a meal high in SFA from Cheddar cheese in overweight and obese individuals with metabolic abnormalities would result in lower circulating pro-inflammatory markers in the postprandial state compared with a meal with equal amounts of SFA from palm oil plus a non-dairy cheese substitute.

## Methods

### Participants

A total of twenty adults (seven men and thirteen women) were recruited. Inclusion criteria were: age 18–65 years, and either a BMI between 25 and 29·9 kg/m^2^ plus two traits of the metabolic syndrome defined by the American Heart Association as waist circumference (WC) > 40 inches (>102 cm) for men and 35 inches (>89 cm) for women, fasting plasma TAG ≥ 150 mg/dl (≥ 1.69 mmol/l), fasting plasma HDL-cholesterol <40 mg/dl (<1.03 mmol/l) for men and <50 mg/dl (<1.29 mmol/l) for women, blood pressure ≥130/85 mmHg, and fasting glucose ≥100 mg/dl (≥5.6 mmol/l)^(^^22^^)^, or obese (BMI between 30 and 39·9 kg/m^2^). Participants’ insulin sensitivity was determined from their fasting plasma glucose and insulin using the homoeostasis model assessment of insulin resistance (HOMA-IR) with 2·6 or greater as a cut-off point of insulin resistance^(^^23^^)^. Participants’ inflammation status was determined with the measurement of fasting circulating CRP concentrations at 3 mg/l or above as associated with increased CVD risk^(^^24^^)^. Exclusion criteria included immune-related diseases, gastrointestinal disorders, cancer, type 2 diabetes, use of over-the-counter anti-obesity agents or corticoid steroid within the last 12 weeks, daily use of anti-inflammatory pain medication, self-reported eating disorder, poor vein assessment determined by the phlebotomist, known allergy or intolerance to study food, vegetarian, consume >1 serving of fish per week, >14 g of fibre per 1000 kcal (4184 kJ)/d, <16:1 of total dietary *n*-6:*n*-3 ratio, >1 % of daily energy as *trans*-fats, initiation of fish, krill, flax, borage or primrose seed oils within the last 12 weeks, dietary supplements of concentrated soya isoflavones, resveratrol, other polyphenols, initiation of statin therapy within the last 12 weeks, >10 % weight loss or gain during the past 6 months, initiation of an exercise programme in the last month, plan to become pregnant in the next 6 months, pregnancy or lactation, recent initiation, change, or cessation of hormonal birth control within the last 12 weeks, and use of tobacco products. To establish fulfillment of the enrolment criteria subjects completed a questionnaire regarding their health history, diet and medication, completed an online FFQ, provided a fasting blood sample for analysis of blood lipids and glucose, and were assessed for weight, height and WC at the screening visit.

The Institutional Review Board of the University of California at Davis approved the study protocol, and all participants gave written informed consent prior to starting the study. The study is registered at clinicaltrials.gov under NCT01803633.

### Study design

**Fig. 1.**
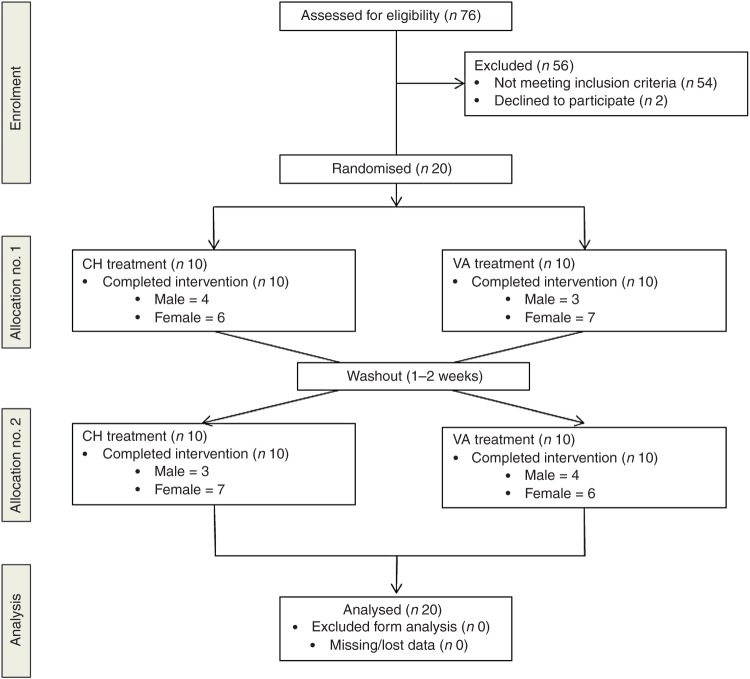
Enrolment and follow up of participants in the randomised cross-over trial. CH, Cheddar cheese treatment; VA, vegan-alternative treatment.

A randomised, cross-over design was used in which investigators were blind to treatment order. All participants received both treatments. To avoid any interaction effects a 1- to 2-week washout period was observed between treatments. Treatment order was randomly assigned using a random allocation sequence generator (randomization.com; seed# 4234) (Fig. 1).

Volunteers were instructed to avoid alcohol consumption, non-steroidal inflammatory drug use, and any anti-inflammatory supplements such as fish, krill, flax, borage or primrose seed oils 72 h prior to each test day. Volunteers were instructed to avoid seafood, and were asked to record everything they consumed 24 h before the test day, not to exercise, and not to consume anything past 20.00 hours. We analysed the 1-d dietary records using the Nutrition Data System for Research (NDSR; University of Minnesota) to ensure that participants complied with pre-study dietary instructions.

After a 10–12 h fast participants arrived at the Western Human Nutrition Research Center. Upon arrival volunteers turned in their 1-d food record, filled out a modified gastrointestinal questionnaire^(^^25^^)^, provided a urine sample and fasted blood draw via venepuncture. Measurements for blood pressure, heart rate, weight and WC were taken. Participants then consumed the high-fat breakfast challenge within 20 min. Postprandial blood draws and urine samples were collected at 1, 3 and 6 h. These time points were chosen based on previous studies demonstrating peak cytokine concentrations observed 3 or 6 h after a high-fat meal challenge^(^^16^^)^ and oxylipins and eicosanoids change at 1 and 6 h from baseline^(^^26^^,^^27^^)^.

Throughout the test day participants were not allowed to consume any food, but could drink bottled water *ad libitum*. Participants either stayed at the research centre or left between blood draws to attend classes or return to work. To minimise physical activity, if participants left the research facility they did so by car, not by foot or bicycle, and returned approximately 15 min and rested for 10 min prior to the next blood draw.

### Dietary challenges

The dietary challenges consisted of grilled sandwiches served with a blended beverage. The cheese (CH) test meal consisted of medium Cheddar cheese (Tillamook Medium Cheddar Cheese) while the vegan alternative (VA) consisted of Daiya Cheddar cheese (Daiya). Both sandwiches were made with 100 % Whole Wheat Sandwich Thins (Oroweat). Each meal was paired with a blended beverage to allow for the matching of energy, total fat, carbohydrates and protein (Supplementary Table S1).

Each of the two meal challenges provided 40 % of the participant's total energy intake. Energy intake was determined by using the National Academy of Sciences equation from the Institute of Medicine Dietary Reference Intake that takes physical activity into account in addition to sex, age, weight and height^(^^28^^)^. Physical activity was determined using the Baecke Physical Activity questionnaire, which assesses habitual physical activity and identifies three components of physical activity: work, sport and leisure time excluding sport^(^^29^^)^.

Each meal consisted of about 50 % fat, about 40 % carbohydrates, and about 10 % protein and delivered between 45 and 75 g of fat depending on the individual's energy intake. The Cheddar cheese provided 44 % of the fat in the CH meal and palm oil provided 38 % of the fat in the non-dairy cheese substitute meal. The meals were isoenergetic, had the same macronutrient composition as well as similar MUFA, PUFA and SFA composition (Table 1). The only difference between the test meals was individual fatty acid composition, the matrix in which it was delivered, dietary cholesterol and Ca concentrations. Participants were instructed to eat everything on their plate and rinse their blended beverage cup with water, then drink the rinse water.

**Table 1.** Nutrient composition of the test meals† (Mean values and standard deviations)

	CH meal		VA meal	
	Mean	sd	Mean	sd
Energy (kcal)	1002·9	114·5	1002·1	113·9
Energy (kJ)	4196·0	478·8	4192·6	476·3
Total carbohydrate				
g	103·7	11·8	105·0	11·9
% total energy	39·3	0·0	39·0	0·0
Total protein				
g	27·8	3·2	28·8	3·3
% total energy	11·0	0·0	10·2	0·0
Total fat				
g	56·6	6·5	57·9	6·6
% total energy	49·6	0·0	50·8	0·0
Total SFA				
g	19·2	2·2	19·7	2·2
% total energy	16·8	0·0	17·2	0·0
Total MUFA				
g	21·1	2·4	21·7	2·5
% total energy	18·6	0·0	19·1	0·0
Total PUFA				
g	13·4	1·5	13·8	1·6
% total energy	11·8	0·0	12·1	0·0
SFA 4 : 0 (butyric acid) (%)***	1·4	0·0	0·0	0·0
SFA 6 : 0 (caproic acid) (%)***	0·7	0·0	0·1	0·0
SFA 8 : 0 (caprylic acid) (%)***	0·4	0·0	0·9	0·0
SFA 10 : 0 (capric acid) (%)	0·8	0·0	0·8	0·0
SFA 12 : 0 (lauric acid) (%)***	0·7	0·0	5·6	0·0
SFA 14 : 0 (myristic acid) (%)***	4·5	0·0	2·5	0·0
SFA 16 : 0 (palmitic acid) (%)***	16·10	0·01	20·24	0·02
SFA 18 : 0 (stearic acid) (%)***	7·71	0·00	3·28	0·00
SFA 20 : 0 (arachidic acid) (%)***	0·00	0·00	0·12	0·00
SFA 22 : 0 (behenic acid) (%)***	0·22	0·00	0·15	0·00
MUFA 16 : 1 (palmitoleic acid) (%)***	1·50	0·00	0·39	0·00
MUFA 18 : 1 (oleic acid) (%)*	34·97	0·02	36·78	0·01
MUFA 20 : 1 (gadoleic acid) (%)***	0·22	0·00	0·37	0·00
PUFA 18 : 2 (linoleic acid) (%)	22·98	0·02	21·80	0·02
PUFA 18 : 3 (linolenic acid) (%)***	0·72	0·00	1·94	0·00
Cholesterol (mg)***	79·20	9·01	0·00	0·00
Total dietary fibre (g)***	12·27	1·39	15·60	1·77
Ca (mg)***	487·11	55·38	129·71	14·75

CH, cheese; VA, vegan alternative.

Significant difference between the two meals: *P < 0·05, **P < 0·005, ***P < 0·0005.

† Comparison of the two dietary challenges. Nutrient composition was obtained using Nutrition Data System for Research (NDSR). Test meals were based on total energy expenditure thus values shown are average of all test meals (*n* 20).

### Blood analyses

Whole blood was collected at the Western Human Nutrition Research Center by a trained phlebotomist for each time point. Whole blood was centrifuged in a tabletop ultracentrifuge for 10 min at 4°C at 1300 ***g*** within 30 min of collection. Plasma was immediately separated into 1·5 ml aliquots, and immediately frozen at –70°C until analysis. Serum was isolated and transferred into 1·5 ml aliquots after clotting for 30 min on ice and centrifugation and immediately frozen at –70°C until analysed.

### Inflammatory markers

Serum collected at 0, 1, 3 and 6 h was used to analyse cytokines (IL-10, IL-17, IL-1β, IL-2, IL-6, IL-8, monocyte chemotactic protein-1 (MCP-1), TNFα), as well as the acute-phase proteins (C-reactive protein (CRP), serum amyloid-A (SAA)), and vascular injury molecules soluble intracellular adhesion molecule (sICAM) and soluble vascular adhesion molecule. Plasma was used to measure IL-18 concentrations. Analysis of all inflammatory markers was completed using an electro-chemiluminescence detection system using multiarray technology (SECTOR Imager 2400; Meso Scale Discovery) according to the manufacturer's instructions. In brief, this system uses multi-array ninety-six-well plates with multi-electrodes per well. Each electrode has a different capture antibody, allowing for the analysis of multiple markers of interest at once. The assay procedure is similar to that of a classic sandwich ELISA. A small amount of sample (25–50 µl) serum or plasma is added to each well. The markers of interest bind to the pre-coated capture antibodies. To complete the ‘sandwich’ a detection antibody that is conjugated with a label is added and binds to the analyte. Finally the addition of a read buffer allows for the activation of the label on the detection antibody, resulting in illumination after electrochemical stimulation via voltage that runs through the plate in the reader. The instrument measures the intensity of the light to quantify a measure of each analyte.

### Metabolic parameters

Plasma glucose, insulin and a lipid panel including TAG, total cholesterol, HDL-cholesterol, LDL-cholesterol, HDL:LDL ratio and non-HDL-cholesterol were assessed by the clinical laboratory at the University of California Medical Center (Sacramento, CA) at baseline and at 1, 3 and 6 h after consuming the high-fat challenge meal.

### Clinical characteristics

Anthropometric data were collected at the screening visit and each test day visit. Measurements included body weight (6002 Wheelchair Scale; Scale-tronix), WC (QM2000 Measure Mate; QuickMedical), height (Ayrton Stadiometer Model S100; Ayrton Corporation), blood pressure and resting heart rate (Carescape V100 with Critikon Dura-cuf for either adults or large adults; GE Medical Instruments). BMI was calculated as kg/m^2^.

### Statistical analysis

This study was a 2 × 4 factorial design: two treatments x four time points. Sample size was calculated based on our primary outcome marker using the means and standard deviations for IL-6 from a similar human study with overweight volunteers^(^^16^^)^. A secondary analysis was also conducted on other pro-inflammatory cytokines, acute-phase proteins and vascular adhesion molecules based on previous research that showed changes in these markers in healthy participants in response to high-fat test meals containing similar amounts of fat as used in our study (for a review, see Herieka & Erridge^(^^30^^)^).

To ensure that our sample size reflected the number of subjects needed to be 95 % confident of our results, with 80 % power we needed eighteen volunteers. To account for attrition 10 % was added for a final sample size of twenty. All data were checked for normality visually with histograms and Q-Q plots as well as numerically with the Shapiro–Wilk test. Data were transformed as needed prior to conducting statistical analysis. For cytokines with <10 % of samples below the detection limit (IL-17 only) the value recorded was the lowest limit of detection provided by the manufacturer and divided by 10. For dependent variables with >10 % of the data below the detection limit these markers were excluded from statistical analysis (IL-1β and IL-2).

Dietary data between treatments were compared using a paired two-tailed *t* test. Differences in baseline concentration between treatments were tested using a paired sample two-tailed *t* test. A mixed linear model was conducted with treatment and time as fixed factors, subjects as the random effect and treatment × time as the interaction term. If time was significant, multiple comparison *post hoc* analysis with Bonferroni correction was carried out to compare the concentrations at 0 *v*. 1 h, 0 *v*. 3 h, 0 *v*. 6 h, 1 *v*. 3 h, 1 *v*. 6 h, and 3 *v*. 6 h.

The incremental AUC (iAUC, area above baseline) and decremental AUC (area below baseline) using the conventional trapezoid method were used to compare postprandial responses between groups (response to test meals)^(^^31^^)^. The iAUC was chosen over the total AUC because it reflects the postprandial rise of metabolite concentrations above the non-zero fasting value^(^^32^^)^. iAUC between CH and VA groups were compared via paired *t* tests and ANCOVA with subject as the random variable, treatment as the fixed factor and baseline CRP concentrations as the covariate.

We conducted secondary analyses to determine if pre-existing clinical conditions affected the inflammatory response to the high-fat meal challenge. We coded subjects for high *v*. low baseline CRP levels known to be associated with increased vascular risk (<3 mg/l, *n* 10; or >3 mg/l, *n* 10). ANCOVA was used to identify statistically significant differences in postprandial inflammatory markers between treatments using baseline values of CRP as the covariate.

Differences were considered significant at *P* < 0·05. Statistical analyses were performed using SPSS version 20.0 software for Macintosh (SPSS).

## Results

### Subject characteristics

A total of seventy-six participants underwent screening. As shown in Fig. 1, twenty subjects were randomly assigned to one of two treatment groups in a cross-over design: CH (*n* 20) and VA (*n* 20). After a 1- to 2-week washout period each subject crossed over to the remaining treatment. All subjects completed both treatment arms with no attrition.

The baseline characteristics of the subjects are shown in Table 2 and metabolic characteristics of each participant are found in Supplementary Table S2. Our study population was predominantly female (65 %) and Caucasian (75 %). With respect to the metabolic syndrome, six of the twenty participants had the metabolic syndrome with three or more of the five diagnostic traits, seven participants had two of the five traits, and seven participants had only one trait. When assessing their metabolic and inflammatory status, fourteen of the twenty participants were considered insulin resistant with a HOMA-IR >2·6; ten of the twenty participants had a high baseline CRP concentration (>3 mg/l); and six of the twenty participants had both a HOMA-IR >2·6 and baseline CRP concentration above 3 mg/l. Additionally, thirteen of the twenty participants had a BMI of 30 kg/m^2^ or greater and were abdominally obese (WC >35 inches (>89 cm) for women and >40 inches (>102 cm) for men).

**Table 2.** Subject baseline characteristics* (Mean values and standard deviations)

	Mean	sd	MetS criteria†	Range
Age (years)	49	11		24–64	
Participants with high CRP (years)‡	50·6	12·3		24–64	
Participants with low CRP (years)‡	47·9	9·4		25–57	
Weight (kg)	90·5	9·5		66·1–109·0	
Height (m)	1·7	0·1		1·5–1·8	
BMI (kg/m^2^)	31·7	2·9		24·4–37·1	
WC (inches)	38·7	2·4		32–42·4	
WC, males (inches)§	39·4	1·3	>40	37·2–41	
WC, females (inches)§	38·3	2·8	>35	32–42·4	
WC (cm)	98·3	6·1		81–107·7	
WC, males (cm)§	100·1	3·3	>102	94·5–104·1	
WC, females (cm)§	97·3	7·1	>89	81·3–107·7	
Systolic BP (mmHg)	127	14·1	≥130	98–151·5	
Diastolic BP (mmHg)	78·1	9	≥85	57–98·5
HDL-cholesterol				
mg/dl	45·9	9·7		31–60
mmol/l	1·19	0·25		0·80–1·55
HDL-cholesterol, male§				
mg/dl	41·9	10·3	<40	31–56
mmol/l	1·08	0·27	<1·03	0·80–1·45
HDL-cholesterol, female§				
mg/dl	48·1	9·0	<50	33–60
mmol/l	1·24	0·23	<1·29	0·85–1·55
Fasting glucose				
mg/dl	91·8	7·4	≥100	78–104
mmol/l	5·1	0·4	≥5·6	4·3–5·8
Fasting TAG				
mg/dl	124·4	45·7	≥150	65–224
mmol/l	1·40	0·52	≥1·69	0·73–2·53

MetS, metabolic syndrome; CRP, C-reactive protein; WC, waist circumference; BP, blood pressure.

* Measurements taken at screening visit (*n* 20).

† MetS as defined by the American Heart Associations.

‡ High baseline CRP *n* 10; low baseline CRP *n* 10.

§ Male *n* 7; female *n* 13.

### Dietary intake

Dietary analysis of the 1-d food record completed prior to each test day revealed no significant differences in consumption of macro- and micronutrients (data not shown).

### Metabolic parameters

Baseline and postprandial changes for metabolic markers (total cholesterol, HDL, LDL, TAG) are shown in Table 3. No time × treatment interaction or main effect of treatment were observed for any of these markers; however, there was a significant difference over time for all markers.

**Table 3.** Concentrations of metabolic parameters before and after dietary challenge (Pooled mean values and standard deviations)*

	Time										
	0 h		1 h		3 h		6 h		*P*		
	Mean	sd	Mean	sd	Mean	sd	Mean	sd	Treatment	Time	Interaction
Total cholesterol									0·87	0·001	0·96
mg/dl	209·0	44·3	215·1	45·2	209·9	42·4	212·2	45·6			
mmol/l	5·41	1·15	5·57	1·17	5·44	1·10	5·50	1·18			
HDL-cholesterol									0·95	0·002	0·51
mg/dl	47·3	10·9	48·3	11·4	45·8	10·0	45·7	10·5			
mmol/l	1·23	0·28	1·25	0·30	1·19	0·26	1·18	0·27			
LDL-cholesterol									0·98	<0·0005	0·24
mg/dl	135·8	38·5	132·4	38·8	126·2	36·4	124·6	41·1			
mmol/l	3·52	1·00	3·43	1·00	3·27	0·94	3·23	1·06			
HDL:LDL	4·6	1·1	4·6	1·0	4·7	1·1	4·7	1·1	0·93	<0·0005	0·38
TAG									0·60	<0·0005	0·67
mg/dl	129·0	44·4	171·2	54·7	199·9	75·2	218·6	77·4			
mmol/l	1·46	0·50	1·93	0·62	2·26	0·85	2·47	0·87			
Non-HDL-cholesterol									0·88	<0·0005	0·59
mg/dl	161·6	41·7	166·7	42·4	164·1	40·3	166·4	43·6			
mmol/l	4·19	1·08	4·32	1·10	4·25	1·04	4·31	1·13			

* Values are pooled means of both treatments since there was no treatment effect.

### Postprandial glycaemia

**Fig. 2.**
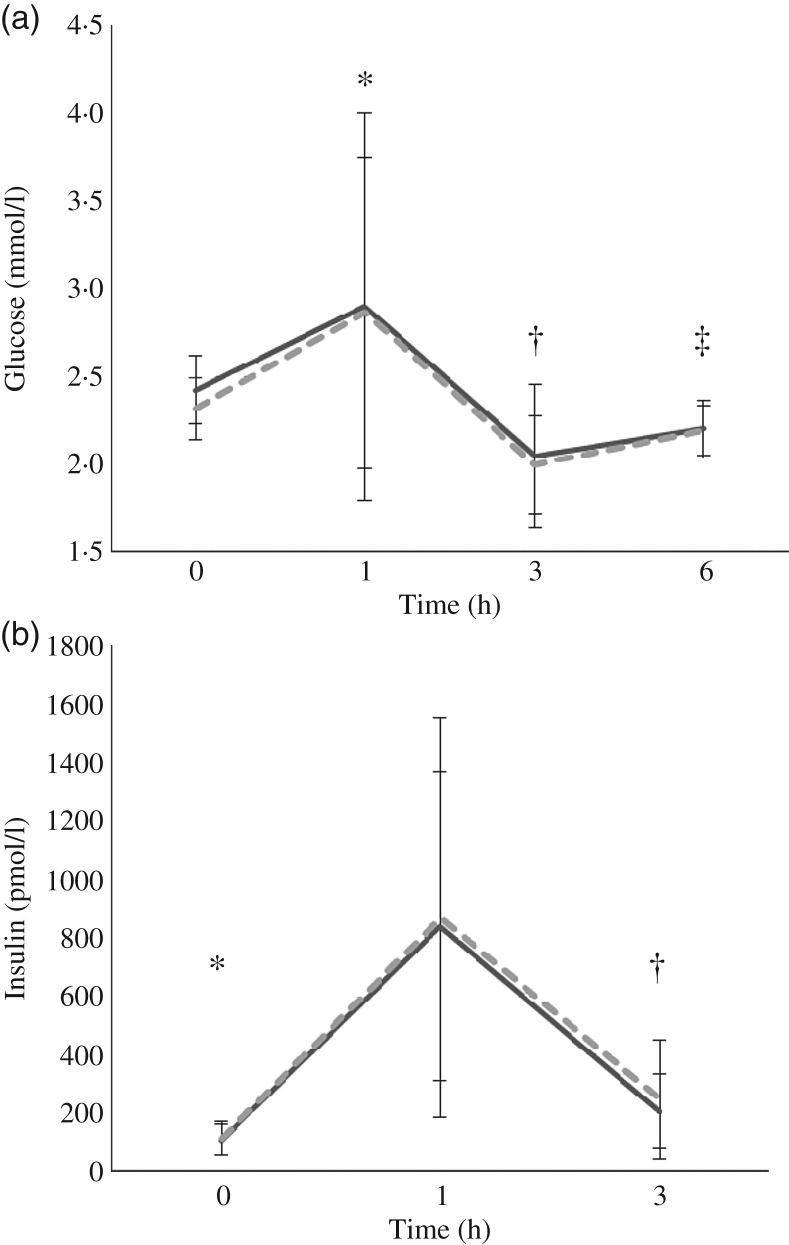
Postprandial response of glucose (a) and insulin (b) before and after high-fat mixed meal rich in SFA from either vegan alternative cheese (---) or Cheddar cheese (––). Values are means, with standard deviations represented by vertical bars (*n* 20). * Significant difference between 0 and 1 h (*P* < 0·0005). † Significant difference between 1 and 3 h (*P* < 0·0005). ‡ Significant difference between 3 and 6 h (*P* = 0·01).

At baseline, insulin concentrations did not differ between test days (*P* = 0·8) while glucose concentrations did (*P* = 0·004); however, since there was no treatment effect this statistical difference was considered a random occurrence. Following each meal, there was a rapid increase in both glucose and insulin from 0 to 1 h (*P* = 0·029 and *P* < 0·0005, respectively) followed by a decrease from 1 to 3 h (*P* < 0·0005 for both) and an increase in glucose from 3 to 6 h (*P* = 0·01). The glucose (*P* = 0·602) and insulin (*P* = 0·908) responses did not differ between the treatments (Fig. 2).

### Postprandial lipaemia

**Fig. 3.**
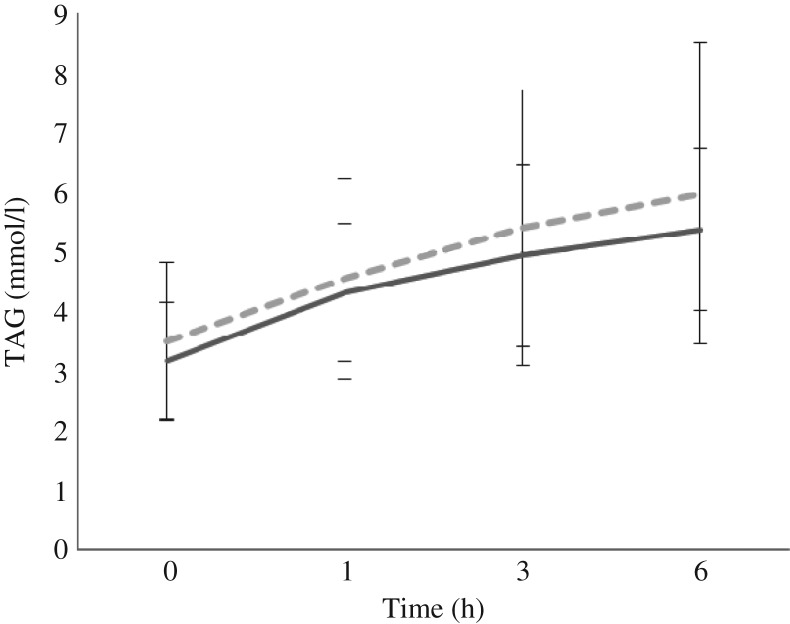
Postprandial response of serum TAG after both vegan alternative (---) and Cheddar cheese (––) meals. Values are means, with standard deviations represented by vertical bars (*n* 20). There was no difference between the treatments but there was a significant increase over time (*P* < 0·05) for all time points: 0–1, 0–3, 0–6, 1–3, 1–6, and 3–6 h.

Fasting/baseline TAG concentrations were comparable between the two treatments (*P* = 0·245). After meal consumption, postprandial TAG concentration rose steadily (*P* < 0·05 for all time points: 0–1, 0–3, 0–6, 1–3, 1–6, and 3–6 h) with no sign of a decrease at the 6 h time point (Fig. 3).

### Inflammatory markers

**Fig. 4.**
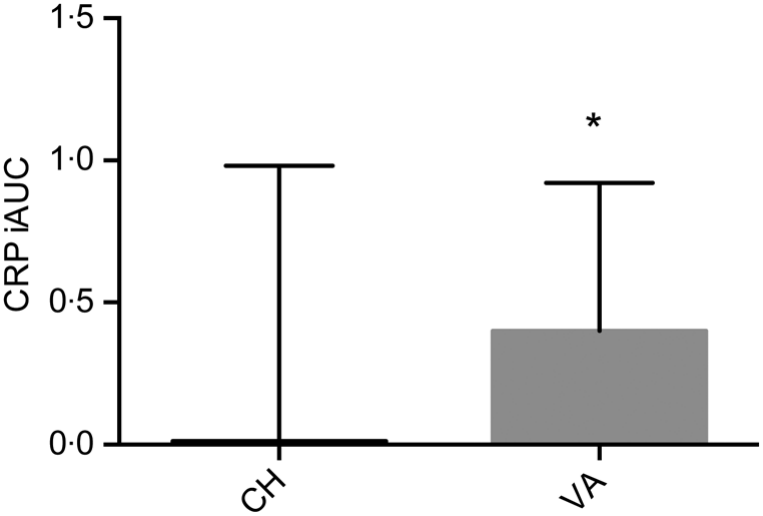
Postprandial serum C-reactive protein (CRP) concentrations over the 6 h postprandial period after the Cheddar cheese (CH) and vegan alternative (VA) meals. Values are means, with standard deviations represented by vertical bars. * The VA meal resulted in a significantly greater overall CRP concentration (*P* = 0·033) when compared with the CH meal. iAUC, incremental AUC.

IL-1β and IL-2 were below the detection limit (for about 70 and 35 % of samples, respectively) and therefore, excluded from the statistical analysis. Similar results for these two particular cytokines have been observed^(^^33^^)^. Baseline concentrations of all markers related to inflammation were comparable between the two interventions. CRP as the iAUC was the only marker with a significant difference between treatments, with the VA meal resulting in a significantly greater overall CRP concentration (*P* = 0·033) when compared with the CH meal (Fig. 4). There was no treatment effect observed for any of the other inflammatory markers. However, multiple markers did demonstrate an effect over time; SAA peaked at 1 h postprandially, IL-8 peaked at 3 h, while IL-6, IL-18, MCP-1 and TNFα peaked at 6 h postprandially (Table 4). Secondary analyses based on baseline CRP values indicated that those with pre-existing fasting CRP concentrations ≥3 mg/l did not have a different response to treatment compared with participants with baseline CRP concentrations <3 mg/l. We conducted a secondary analysis using baseline CRP concentrations as a stratification and found that volunteers who had a baseline CRP value >3 mg/l had significantly higher concentrations of IL-6, SAA and sICAM at each time point compared with the subjects who had baseline CRP values <3 mg/l.

**Table 4.** Concentrations of inflammatory markers before and after the dietary challenge (Pooled mean values and standard deviations)*

	Time											
	0 h		1 h		3 h		6 h		*P*				
	Mean	sd	Mean	sd	Mean	sd	Mean	sd	Treatment	Time	Interaction	LLOD†
IL-6 (pg/ml)	0·94	0·64	0·79	0·63	0·94	0·77	1·16	1·15	0·818	0·001	0·146	0·06
IL-8 (pg/ml)	8·46	2·60	9·06	2·51	9·47	2·46	8·48	2·63	0·97	0·004	0·866	0·04
IL-10 (pg/ml)	6·94	17·99	6·69	16·44	7·14	18·73	7·30	18·13	0·989	0·305	0·459	0·03
IL-17 (pg/ml)	3·08	4·35	3·11	4·89	3·40	4·71	4·10	7·62	0·889	0·681	0·155	0·74
IL-18 (pg/ml)	35·85	23·24	38·63	25·24	37·05	24·17	39·35	23·43	0·958	0·001	0·217	0·71
TNFα (pg/ml)	1·95	0·82	1·99	0·79	2·03	0·79	2·07	0·89	0·836	0·001	0·267	0·04
MCP-1 (pg/ml)	456·7	238·5	496·8	228·2	498·4	246·5	521·8	238·1	0·645	0·001	0·599	0·09
CRP (mg/l)	4·57	5·00	4·72	5·32	4·46	5·42	4·52	5·21	0·588	0·256	0·414	1·33 × 10^−6^
SAA (mg/l)	9·21	8·92	9·51	9·48	9·13	10·12	8·52	9·44	0·473	0·023	0·361	1·09 × 10^−5^
sICAM (mg/l)	0·28	0·09	0·30	0·16	0·29	0·14	0·27	0·09	0·629	0·427	0·834	1·03 × 10^−6^
sVCAM (mg/l)	0·42	0·10	0·45	0·22	0·46	0·20	0·42	0·13	0·637	0·317	0·467	6 × 10^−6^

LLOD, lower limit of detection; MCP-1, monocyte chemotactic protein-1; CRP, C-reactive protein; SAA, serum amyloid-A; sICAM, soluble intracellular adhesion molecule; sVCAM, soluble vascular adhesion molecule.

* Values are pooled means of both treatments since there was no treatment effect.

† Values from Meso Scale Discovery.

## Discussion

In this study, more than half (55 %) of the measured serum inflammatory markers (IL-6, IL-8, IL-18, TNFα, MCP-1 and SAA) significantly changed over the 6 h postprandial period from baseline. Some of these findings are consistent and some are not consistent with previous findings. Based on the literature we had expected IL-6 to increase after the high-fat challenges and peak around the 3 h time point, which has previously been observed in men with the metabolic syndrome^(^^34^^)^, overweight, obese men and women^(^^16^^,^^35^^,^^36^^)^, and healthy participants^(^^37^^,^^38^^)^. However, to our surprise, the results presented here showed an initial decrease in serum IL-6 concentration from baseline to the 1 h time point after consuming both high-fat meals, followed by a significant rise at 3 h. Manning *et al*.^(^^36^^)^ reported similar findings of a temporary decrease in IL-6 at 1 h after five test meals rich in cream, olive oil, rapeseed oil, potato or All-Bran in obese and healthy women, suggesting this might be due to the anti-inflammatory effects of insulin^(^^39^^)^. Our results support this, as insulin peaked at 1 h, the time at which IL-6 concentrations were lowest.

IL-8 has not been commonly included in high-fat postprandial inflammation studies^(^^30^^)^; however, one postprandial study found no change over time in overweight men^(^^16^^)^ or in healthy or obese women^(^^36^^)^. IL-8 plays an important role in atherosclerosis in part by stimulating the production of other pro-inflammatory cytokines such as TNFα and IL-1β^(^^40^^)^. Our results showed an initial rise in IL-8 concentrations after both treatments with a peak at the 3 h time point. An increase in IL-18 has been linked to CVD as it is a powerful cytokine whose functions include the production of pro-inflammatory cytokines such as TNFα, IL-1β, sICAM and soluble vascular adhesion molecule^(^^41^^)^. We are one of the first groups to analyse the postprandial response of IL-18 after a high-fat challenge. Our results showed a significant increase in IL-18 after both high-fat treatments, complemented by a similar increase in TNFα.

TNFα, a cytokine commonly measured in the postprandial state, significantly increased after consumption of a large lipid load and peaked at 6 h. These data are consistent with several reports in healthy, type 2 diabetic, overweight, and obese men and women after high-fat test meals^(^^15^^,^^16^^,^^35^^,^^36^^,^^38^^)^. These data may be explained by a greater number of monocytes in circulation after a high-fat meal^(^^42^^)^ and the rise in IL-8 and IL-18 at 3 h. While we do not have data on lymphocyte count for this study, it would be of interest for future studies to incorporate this analysis. Our results showed a significant increase in MCP-1 from baseline at 1, 3 and 6 h after both meals. Similar results have been reported previously in patients with the metabolic syndrome who consumed a high-saturated fat meal^(^^43^^)^. However, not all studies have observed this postprandial increase. Masson & Mensink^(^^16^^)^ found a significant decrease in serum MCP-1 in overweight men after both high-fat meals that provided a total of 50 g of fat (butter *v*. linoleic acid). In our study, the majority of the participants were women and fat in the test meals was provided as 50 % of each individual's total energy expenditure and ranged from 45 to 75 g. It is not known if the total amount of fat consumed or sex influence MCP-1 responses in the postprandial period.

SAA, an acute-phase protein that is primarily synthesised by the liver, increases several-fold during the inflammatory response to tissue injury^(^^44^^)^. Its expression is stimulated by cytokines (IL-6, IL-1β, TNFα)^(^^45^^)^; however, SAA also stimulates the secretion of IL-6 in connective tissue^(^^46^^)^. SAA circulates in the blood as part of HDL particles. In this complex, SAA mediates the binding of HDL to proteoglycans, promoting oxidation of the lipoprotein which impairs its ability to remove cellular cholesterol, leading to cholesterol accumulation in macrophages and foam cell formation^(^^47^^)^. Based on SAA's ability to stimulate release of IL-6 by some tissues, we expected to see a postprandial increase after the high-fat meals. Instead, our results indicate a significant decrease from baseline at the 6 h time point similar to the decline in HDL-cholesterol over the postprandial period. A similar decline in SAA concentration was observed by Esser *et al*.^(^^48^^)^ after providing healthy men a high-fat meal, while an increase was seen after the same volunteers consumed a lower-fat standard breakfast.

When the total concentration of each analyte over the 6 h postprandial time was calculated as the iAUC, only CRP was different between the two test meals, with the VA meal resulting in a significantly greater overall CRP concentration (*P* = 0·033) when compared with the CH meal. It is unknown what components in cheese could reduce CRP concentration or what components in the vegan test meal could stimulate the increase in CRP concentration over the 6 h postprandial time period. Schmid *et al*.^(^^49^^)^ recently reported no difference in postprandial CRP after consumption of a high-fat control or high-fat control with milk in normal-weight, overweight and obese men. CRP is an acute-phase protein that is synthesised by the liver and increases several-fold during the inflammatory response to tissue injury^(^^44^^)^ and responds to diet and exercise^(^^50^^,^^51^^)^. Its release by the liver is stimulated by IL-6 and alternatively, CRP also induces secretion of IL-6 by endothelial cells^(^^52^^)^. There are inconsistent findings on the effects of a high-fat test meal on circulating CRP concentrations as a result of the composition of the test meals used, target populations and timings of blood draws. For example, in lean individuals, plasma CRP measured 24 h after consumption of a high-fat breakfast was significantly lower from baseline when the breakfast contained a high *v*. low saturated:unsaturated fat ratio^(^^15^^)^. Other reports have shown that CRP does not change within 6 h after consumption of high-fat mixed meals in lean, overweight and abdominally obese participants^(^^15^^,^^31^^,^^35^^,^^49^^)^. However, in type 2 diabetic participants, CRP significantly increased from baseline within 4 h in response to a high-fat test meal^(^^53^^,^^54^^)^. In our study, the postprandial CRP response to the test meal could be explained by the interaction between metabolic phenotype and the test meal^(^^31^^)^. Perez-Martinez *et al*.^(^^31^^)^ recently reported higher postprandial plasma CRP to a high-fat meal in metabolically abnormal subjects compared with normal, overweight and obese, metabolically healthy patients. In our study, the majority of our participants were metabolically abnormal according to elevated circulating CRP (>3 mg/l), insulin resistance (HOMA-IR > 2·6) and abdominal adiposity, yet we did not have enough statistical power to determine the relationship between the test meal and metabolic phenotype.

In our study, participants had slightly elevated insulin concentrations at baseline as well as high total and LDL-cholesterol levels, which were expected based on the inclusion criteria. As expected, both high-fat challenges significantly increased plasma TAG concentrations postprandially; however, the concentrations surprisingly peaked at 6 h. In metabolically healthy individuals, the peak in plasma TAG after a high-fat mixed meal occurs at about 4 h and declines by 6 h^(^^49^^,^^55^^)^. Because 70 % of our study participants were insulin resistant, it is possible that the plateau in plasma TAG at 6 h is a result of their insulin resistance. These data are supported by the report that the postprandial plasma TAG concentration was found to plateau or peak at 6 h in individuals with pre-diabetes and type 2 diabetes, after consumption of a mixed meal containing 62 g fat^(^^55^^)^.

Postprandial lipaemia has been shown to be strongly associated with increased risk for coronary artery disease^(^^3^^)^; however, while results from our study suggest that the consumption of a meal enriched in SFA whether from Cheddar cheese or plant fat was sufficient to cause postprandial lipaemia, the postprandial cytokine response in this subject pool was within the normal range. Other studies have found that similar amounts of fat fed in mixed meals did induce a lipaemic response and elicited postprandial inflammation^(^^16^^,^^36^^)^. In our study, the amount of fat used was high, with a mean of 59 g and ranged 45–75 g scaled to each individual's energy intake. Thus, we expected that inflammatory responses would increase from baseline. It is possible that participants in this study had delayed changes in postprandial inflammatory markers given that their TAG levels were still increasing and possibly had not peaked yet by 6 h.

Cross-sectionally, dairy product consumption has been shown to be inversely correlated with inflammatory markers among healthy adults^(^^56^^)^. There are many possibilities to explain how the myriad components in dairy products, their interactions and synergy upon digestion and metabolism could influence postprandial inflammation. Dairy components that are reported to confer anti-inflammatory effects include dairy proteins and peptides^(^^57^^)^; SCFA^(^^11^^)^ as trophic factors for intestinal cells^(^^58^^)^; starter and non-starter bacteria in fermented dairy products^(^^12^^)^; oligosaccharides as prebiotics^(^^59^^)^; and milk fat globule membrane and its proteins and polar lipids^(^^13^^)^. The effect of a meal composed of the dairy matrix as Cheddar cheese on postprandial inflammation compared with a non-dairy cheese substitute enriched with SFA in the form of palm oil has not been investigated. Palm oil was chosen as the source of SFA for the non-dairy cheese alternative arm because it is commercially available, widely consumed around the world and provides a similar SFA composition to dairy fat compared with other plant-based fats.

The lack in finding a ‘treatment’ effect to the high-fat test meals for several of the inflammatory markers could be explained by several limitations. First, this study was powered for postprandial changes in serum IL-6. Our sample size was not large enough to permit statistical comparisons among different metabolic phenotypic groups (i.e. high *v*. low CRP; insulin resistant *v*. sensitive, with or without the metabolic syndrome). In our study, seven participants were diagnosed with the metabolic syndrome and of the thirteen individuals without classical metabolic syndrome, most had some metabolic abnormalities: five had elevated plasma CRP, nine were insulin resistant, and ten had abdominal adiposity. Abdominal obesity is associated with a pro-inflammatory state and shares features with the metabolic syndrome (for a review, see Després & Lemieux^(^^60^^)^), and 70 % of the participants had abdominal adiposity, yet, we did not quantitatively differentiate between visceral *v*. subcutaneous obesity. Additionally, the lack of significance may also result from the timing of blood collections against the normal diurnal rhythm of inflammatory markers throughout the day^(^^61^^)^. We did not design the collection of time points to match the diurnal patterns of serum inflammatory markers since these patterns vary for each inflammatory marker. Yet, we did ensure that each time point was matched between the two test meals to control for the time of day. While we observed a time effect for some of the inflammatory markers it is important to note that all of these changes fell within the normal range for each marker; thus the observed changes are probably fluctuations around baseline^(^^34^^)^. Furthermore, one possible explanation for the lack of a treatment effect in all inflammatory markers measured is that the serum TAG had not yet peaked. Another limitation of the study is that the test meals were not balanced for dietary Ca in each treatment. The increased dietary Ca found in the CH meal could have formed Ca soaps and thus lowered the amount of fatty acids that were absorbed. However, while some studies found an effect of Ca from cheese on faecal fat excretion^(^^62^^)^, others did not^(^^63^^)^. Moreover, it would have been of interest to increase the sample size to allow equal numbers of men and women in the study population as little is known about the sex difference in inflammation. One study testing the inflammatory effect of a high-fat meal in overweight men and women found IL-6 to be significantly more elevated in women than men^(^^64^^)^. Finally, it is not well known if postprandial inflammation is influenced by the hormonal changes associated with the menstrual cycle as has been shown with fasting circulating lipids^(^^65^^)^. However, one study found that the variation contributed by the postprandial period on circulating TAG and inflammatory mediators was greater than the hormonal differences between the follicular and luteal phases^(^^66^^)^.

## 

### Conclusions

Results from this study provide important information about the specific effects of the dairy-derived matrix *v*. non-dairy alternative SFA as part of a mixed meal on a wide array of inflammatory markers in the postprandial state, directly addressing several key concerns and gaps in knowledge addressed by a recent expert panel. There was no evidence from this study in overweight and obese adults that there is a difference in inflammatory response after a high-fat mixed meal where the predominant fat source is cheese *v*. palm oil. From the inflammatory markers that were measured only IL-6, IL-8, IL-18, MCP-1, TNFα and SAA showed a postprandial response with respect to early postprandial cytokine induction, regardless of which treatment was consumed. When analysed as the iAUC, postprandial CRP was significantly lower in response to the cheese compared with the vegan test meal. Based on these results we conclude that while dietary recommendations encourage a decreased consumption of saturated fat to minimise CVD risk, saturated fat in the form of cheese lowers postprandial inflammation compared with plant sources of saturated fat.

## Supplementary material

For supplementary material accompanying this paper visit http://dx.doi.org/10.1017/jns.2015.40. click here to view supplementary material
