# Supplementary material for: Consumption of a high-fat meal containing cheese compared with a vegan alternative lowers postprandial C-reactive protein in overweight and obese individuals with metabolic abnormalities: a randomised controlled cross-over study
Source: J Nutr Sci. 2016 Feb 9;5:e9. doi: 10.1017/jns.2015.40 (PMC4791521; doi:10.1017/jns.2015.40)
Supplement: Supplementary file 1 [file S2048679015000403sup.zip › S2048679015000403sup001.doc]

**Supplementary Table S1.** Test meal ingredient list

| **Ingredient** | **CH^1^ meal** | **VA^2^ meal** |
| --- | --- | --- |
| **Sandwich** |  |  |
| 100% whole wheat Sandwich Thins (Orowheat, Horsham, PA, USA) | ✓ | ✓ |
| Medium Cheddar Cheese (Tillamook, Tillamook, OR, USA) | ✓ |  |
| Vegan Cheddar Cheese (Daiya, Vancouver, BC, Canada) |  | ✓ |
| **Blended Beverage** |  |  |
| High Oleic Sunflower Oil (Spectrum, Boulder, CO, USA) | ✓ | ✓ |
| High PUFA Sunflower Oil (Hain Pure Foods, Boulder, CO, USA) | ✓ | ✓ |
| Canola Oil (Spectrum, Boulder, CO, USA) |  | ✓ |
| DI Water (Davis, CA, USA) | ✓ | ✓ |
| Polycose (Abott Nutrition, Columbus, OH, USA) | ✓ | ✓ |
| Cream of Tartar (Safeway Select, Pleasanton, CA, USA) | ✓ | ✓ |
| Palm Oil Shortening (Spectrum, Boulder, CO, USA) |  | ✓ |
| Rice Protein (NutriBiotic, Lakeport, CA, USA) |  | ✓ |
| Raspberry Sorbet (Cioa Bella, Florham Park, NJ, USA) | ✓ | ✓ |

The two test meals were isocaloric. Blended beverage was used to closely match protein, carbohydrate, fat (SFA, PUFA, MUFA) composition.

^1^Cheddar Cheese meal

^2^Vegan Alternative meal
