# Supplementary material for: Consumption of a high-fat meal containing cheese compared with a vegan alternative lowers postprandial C-reactive protein in overweight and obese individuals with metabolic abnormalities: a randomised controlled cross-over study
Source: J Nutr Sci. 2016 Feb 9;5:e9. doi: 10.1017/jns.2015.40 (PMC4791521; doi:10.1017/jns.2015.40)
Supplement: Supplementary file 1 [file S2048679015000403sup.zip › S2048679015000403sup002.doc]

**Supplementary Table S2.** Metabolic status of each study participant

| **Subject ID** | **# MetSyn Traits** | **Fasting CRP > 3 mg/L** | **HOMA IR > 2.6** | **Gender** | **Age** | **BMI** | **Waist circumference > 35 inches women or > 40 inches men** | **SBP ≥ 130 mmHg Or DBP ≥ 85 mmHg** | **Fasting Glucose ≥ 100 mg/dL** | **HDL-C < 50 mg/dL women or < 40 mg/dL men** | **Fasting TG ≥ 150 mg/dL** |
| --- | --- | --- | --- | --- | --- | --- | --- | --- | --- | --- | --- |
| 3003 | 1 | X |  | F | 38 | 32.1 | X |  |  |  |  |
| 3005 | 1 |  | X | M | 54 | 31.3 | X |  |  |  |  |
| 3006 | 1 |  | X | F | 54 | 30.4 | X |  |  |  |  |
| 3007 | 1 |  | X | F | 53 | 31.6 | X |  |  |  |  |
| 3008 | 1 |  | X | M | 56 | 29.7 | X |  |  |  |  |
| 3017 | 1 | X | X | F | 62 | 35.2 | X |  |  |  |  |
| 3001 | 2 | X | X | M | 50 | 29.7 |  | X |  | X |  |
| 3002 | 2 |  |  | F | 56 | 26.8 |  | X | X |  |  |
| 3010 | 2 |  |  | M | 25 | 29.3 |  |  |  | X | X |
| 3013 | 2 |  | X | F | 57 | 32.3 | X | X |  |  |  |
| 3016 | 2 | X | X | F | 57 | 35.1 | X |  |  | X |  |
| 3018 | 2 |  | X | M | 37 | 29.5 | X | X |  |  |  |
| 3020 | 2 | X |  | M | 64 | 28.0 | X | X |  |  |  |
| 3004 | 3 | X | X | F | 54 | 37.1 | X |  |  | X | X |
| 3009 | 3 | X |  | F | 41 | 37.0 | X | X |  | X |  |
| 3011 | 3 |  | X | F | 45 | 28.4 | X |  |  | X | X |
| 3012 | 3 |  | X | F | 52 | 33.1 | X | X |  | X | X |
| 3014 | 3 | X | X | M | 49 | 30.0 | X | X |  | X |  |
| 3015 | 3 | X |  | F | 55 | 33.0 | X | X |  | X |  |
| 3019 | 3 | X | X | F | 24 | 33.8 | X |  |  | X | X |

Metabolic syndrome (MetS) defined by the American Heart Association as waist circumference (WC) > 40 inches for men and 35 inches for women, fasting plasma triglyceride (TG) ≥ 150 mg/dL, fasting plasma high density lipoprotein cholesterol (HDL-C) < 40 mg/dL for men and < 50 mg/dL for women, blood pressure (BP) ≥ 130/85 mmHg, and fasting glucose ≥ 100 mg/dL.
